# Effect of Coffee Thermocycling on Color Stability and Translucency of CAD‐CAM Polychromatic High Translucent Zirconia Compared With Lithium Disilicate Glass Ceramic

**DOI:** 10.1002/cre2.918

**Published:** 2024-07-05

**Authors:** Ratchaphat Khomprang, Jeerapa Sripetchdanond, Wareeratn Chengprapakorn

**Affiliations:** ^1^ Esthetic Restorative and Implant Dentistry Program, Faculty of Dentistry Chulalongkorn University Bangkok Thailand

**Keywords:** ceramic materials, optical properties, thermocycling, zirconia

## Abstract

**Aims and Objectives:**

To evaluate the effect of coffee thermocycling on color stability and translucency of CAD‐CAM polychromatic high translucent zirconia compared with lithium disilicate glass ceramic.

**Methods:**

Sixteen rectangular plates (14 × 16 × 1.0 mm) of two ceramic materials (IPS E.max CAD (IEC), IPS E.max ZirCAD Prime [IZP]) were prepared. Each specimen was measured for color coordinates using a spectrophotometer following 30,000 cycles of coffee thermocycling. CIELAB formula was used to determine color and translucency differences (Δ*E* and ΔTP). The means of ΔE and ΔTP were compared using independent samples *t*‐test and were evaluated using their respective 50%:50% perceptibility and acceptability thresholds (PT and AT). One‐way analysis of variance was performed to evaluate the translucency parameter (TP) and surface roughness (Ra) of each material.

**Results:**

Mean Δ*E* values of IEC (4.69) and IZP (4.64) were higher than the AT (Δ*E* ≤ 2.7) with no significant difference found between the two groups (*p* = 0.202). Considering the TP, only IEC showed a statistically significant increase in TP value (*p* < 0.001). However, the mean ΔTP of IEC (3.25) remained within the range of acceptability (1.3 < ΔTP ≤ 4.4).

**Conclusions:**

Within the limitations of this current study, the color stability of all materials was clinically affected by coffee thermocycling. In terms of translucency, only lithium disilicate glass ceramic was influenced by coffee thermocycling. High translucent zirconia had superior translucency stability compared to lithium disilicate glass ceramic.

## 1 Introduction

The esthetic outcome of anterior restorations is becoming a bigger issue in modern society. Lithium disilicate glass ceramic and high translucent zirconia are recommended as materials of choice to restore anterior teeth (Zhang and Lawn 2018; Aslan, Uludamar, and Özkan 2019; de Araújo‐Júnior, Bergamo, and Bastos 2022; El‐Mowafy, El‐Aawar, and El‐Mowafy 2018; Gehrt et al. 2013; Reale Reyes et al. 2023; Wolfart et al. 2009; Teichmann et al. 2017; Dangra and Gandhewar 2014). At present, both types of these ceramics are frequently used together in the case of multiple anterior restorations, such as crowns or veneers. Consequently, shade matching becomes more challenging to achieve an esthetic outcome that closely resembles the shade and translucency of natural teeth (Alnusayri et al. 2022; Agrawal and Kapoor 2013; Fondriest 2003).

All ceramic materials can be fabricated by using a multilayering technique, which consists of translucent feldspathic veneers and opaque core material (Ban 2020; Farhan et al. 2014) to mimic the appearance of natural teeth. However, a major problem of this technique is porcelain chipping (Pjetursson et al. 2021; Spitznagel et al. 2022; Zhang and Kelly 2017; Pereira et al. 2023; Silva et al. 2017; Lohbauer, Scherrer, and Della Bona 2017; Geminiani et al. 2010; Ghodsi and Jafarian 2018). Nowadays, monolithic restorations can be used in a variety of clinical scenarios and may need fewer fabrication steps than multilayered restorations (Ban 2020; Kontonasaki et al. 2019). The possibility of porcelain chipping in all ceramic restoration can be substantially reduced (Kontonasaki et al. 2019; Pereira et al. 2023; Prott et al. 2021). Moreover, the need for tooth preparation is minimized since there is no requirement to create space for the veneering porcelain. In 2011, high translucent tetragonal zirconia polycrystal (TZP) stabilized with 3 mol% of yttria containing less than 0.05 wt.% of alumina (3Y) was introduced to the field of dentistry (Ghodsi and Jafarian 2018). Primarily, 3Y‐TZP is employed to create monolithic zirconia restorations for the posterior region. Later, in 2014, partially stabilized zirconia (PSZ) stabilized with about 5 mol% of yttria (5Y) was utilized in dentistry. 5Y‐PSZ is typically used for the fabrication of monolithic zirconia in the anterior esthetic region because of its ultra translucency, which approaches that of lithium disilicate glass ceramic. In other words, 5Y‐PSZ exhibited considerably higher translucency compared to 3Y‐TZP (Zhang and Lawn 2018). Currently, translucent zirconia can be made from one zirconia generation or by combining several zirconia generations into the same block (Kolakarnprasert et al. 2019).

A newly developed polychromatic high translucent zirconia material that combines several zirconia generations is produced using Gradient Technology (GT), a novel manufacturing method that combines 3Y and 5Y oxide‐ceramics for the highest level of strength and esthetic that closely mimics the dentinoenamel structure of natural teeth. The blocks or discs have natural color transitions and a smooth appearance. According to the manufacturer's data, it has been proposed to replace lithium disilicate glass ceramic.

Few studies have explored the outcome of coffee thermocycling on the recent polychromatic high translucent zirconia and lithium disilicate glass ceramic. Coffee is a widely cherished beverage globally and a primary caffeine source for many students and professionals, which continues to be a vital component of the daily routine in the society (Menke 2018). Many patients have expressed worries about the lasting color durability of their dental restorations when they consume coffee every day. Therefore, the aim of this research was to evaluate the effect of coffee thermocycling on the color stability and translucency of CAD‐CAM polychromatic high translucent zirconia in comparison to lithium disilicate glass ceramic.

The first null hypothesis states that coffee thermocycling does not affect the color of CAD‐CAM polychromatic high translucent zirconia and lithium disilicate glass ceramic. The second null hypothesis states that coffee thermocycling does not influence the translucency of CAD‐CAM polychromatic high translucent zirconia and lithium disilicate glass ceramic.

## 2 Materials and Methods

### 2.1 Specimen Preparation

The specimens were sliced from the CAD‐CAM specimen blocks and discs (Table 1); for IEC, IZP to obtain eight rectangular plates for each ceramic group at an average size of 14 × 16 mm and thickness of 1.0 ± 0.1 mm according to the manufacturer's recommendations. A2 shade was selected for all materials with glazed surface treatment. To ensure standardization, all specimens' dimensions were controlled using a digital micrometer (RS PRO External Micrometer; RS Component Limited, Bangkok, Thailand). The specimens underwent ultrasonic cleaning in distilled water for 15 min. The pre‐crystallized ceramic specimens of lithium disilicate glass ceramic (IEC) were fired for crystallization in furnaces (Programat P300; Ivoclar Vivadent, Schann, Liechtenstein); the polychromatic high translucent zirconias (IZP) underwent a complete sintering process in a ceramic furnace (Infire htc speed; Dentsply Sirona, Charlotte, NC, USA) to enhance their mechanical characteristics, following the manufacturers' guidelines. For the glazing procedure, the specimens were sandblasted with a sandblasting unit (Basic eco; Renfert, Hilzingen, Germany) using aluminum oxide particles (50 μm) for 10 s at 10 mm distance under 2.5 bars pressure. After that, the specimens underwent a 15‐min ultrasonic cleaning in distilled water. Then, a fine coating of glaze material was administered to one side of each specimen using Ivocolor Glaze paste (Ivoclar Vivadent, Schann, Liechtenstein) and fired following the manufacturer's instruction.

**Table 1:** Type of materials.

Materials	Brand	Manufacturer	Compositions
Lithium disilicate glass ceramic	IPS E.max CAD (IEC)	Ivoclar Vivadent, Schann, Liechtenstein	57%–80% SiO_2_, 11%–19% Li_2_O, 0%–13% K_2_O, 0%–11% P_2_O_5_, 0%–8% ZrO_2_, 0%–8% ZnO, 0%–12% Other and coloring oxides
Polychromatic high translucent zirconia	IPS E.max ZirCAD Prime (IZP)	Ivoclar Vivadent, Schann, Liechtenstein	88.0%–95.5% ZrO_2_, >4.5%–≤7.0% Y_2_O_3_, ≤5.0% HfO, 2 ≤ 1.0% Al_2_O_3_, other oxides ≤1.5%.
Glazing material	Ivocolor Glaze paste	Ivoclar Vivadent, Schann, Liechtenstein	Alkali aluminosilicate glass, Solvent, pigments

All procedures for specimen preparation were carried out by the same operator. The specimens were placed in distilled water at 37°C for 24 h before the initial spectral radiance measurement was taken.

### 2.2 Color Measurement

**Figure 1:**
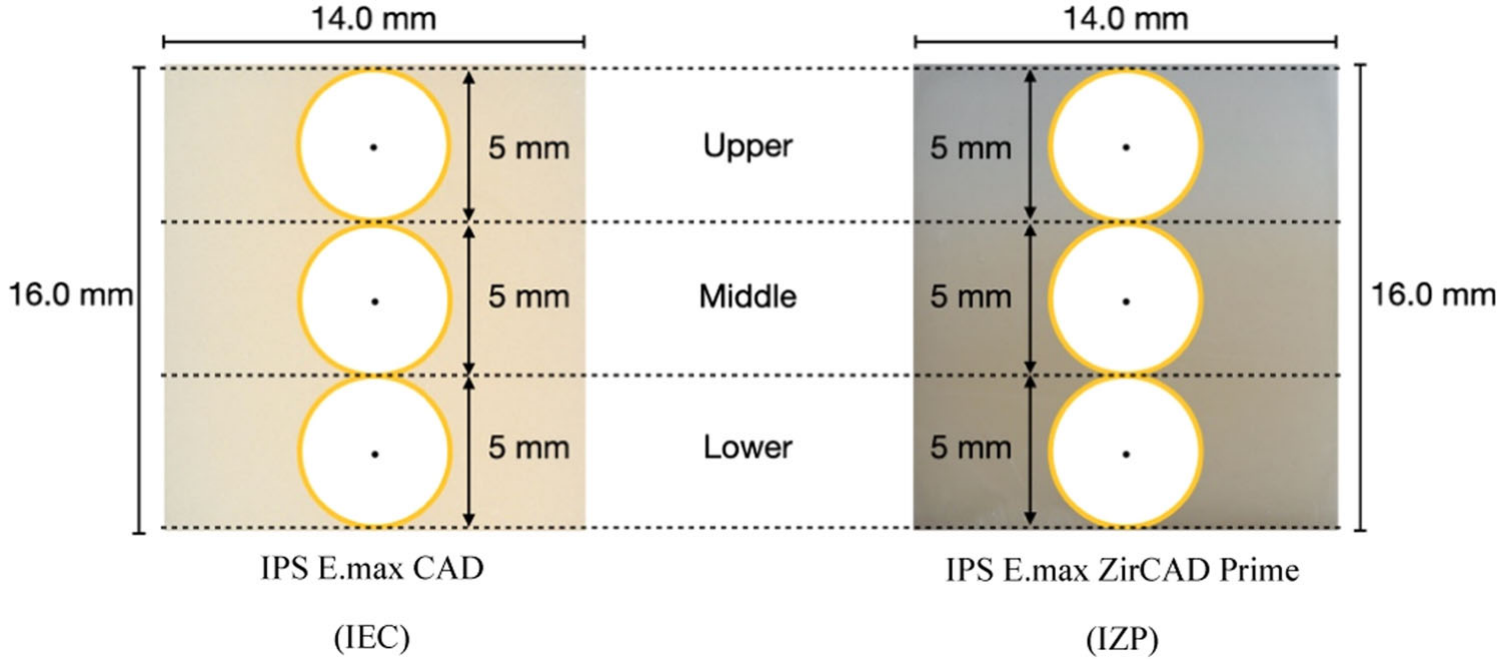
Area of color measurement in each ceramic specimen.

CIE L*a*b* or CIELAB color system, which was defined by the Commission Internationale D'Eclairage (CIE), was used to assess specimen color in this study. Initial color coordinates of all specimens were measured using a spectrophotometer (VITA Easyshade Compact; Vident, A VITA Company, California, USA) on a white background and black background (Sen and Us 2018). The standard black background (CIE L* = 25.3, a* = 1.4, b* = 2.9) and white background (CIE L* = 44.8, a* = 0.2, b* = 2.2) plates were used to calibrate the spectrophotometer before each measurement. The area of measurement in each specimen was equally divided into the upper part, middle part, and lower part (Figure 1). The mean CIE L*a*b* values were obtained following three measurements at the center of each part. The average value was taken on each background with a 5 mm aperture. The same operator made all the measurements.

Color stability or color change is determined by calculating Delta *E* (ΔE*) against a black background using L*a*b* values. The following formula is used to determine the total color differences (Cie.co.at 2022):

$$\mathrm{\Delta E}*\;={\left[{\left(\mathrm{\Delta L}*\right)}^{2}+{\left(\mathrm{\Delta a}*\right)}^{2}+{\left(\mathrm{\Delta b}*\right)}^{2}\right]}^{1/2}.$$

where L* is lightness, a* is green (−a) and red (+a) axis and b* is blue (−b) and yellow (+b) axis. All specimens were measured CIE L*a*b* color coordinates against a black background at the baseline (T0). The perceptibility threshold (PT) of 1.2 and acceptability threshold (AT) of 2.7 were used for the evaluation of color difference in the study (Paravina, Pérez, and Ghinea 2019). PT represents the smallest color difference that can be noticed by 50% of the observers, while AT represents the smallest color difference clinically acceptable for 50% of the observers.

The translucency parameter (TP) was calculated as the color difference between a specimen over standard white and black backgrounds using the following TP formula (Ledić et al. 2015):

$$\mathrm{TP}={\left[{\left(L*W-L*B\right)}^{2}+{\left(a*W-a*B\right)}^{2}+{\left(b*W-b*B\right)}^{2}\right]}^{1/2}.$$

TP: translucency parameter (0–100), a higher TP value indicates more translucent material (Della Bona, Nogueira, and Pecho 2014) L* represents lightness, a* represents the red‐green axis, b* represents the yellow‐blue axis, B: color coordinates over the black background, W: color coordinates over the white background.

Measurement of each sample's final color was made using a spectrophotometer after experiment at the following aging cycles: 10,000 thermocycles (T1), 20,000 thermocycles (T2), and 30,000 thermocycles (T3).

The differences in ΔTP in this study were calculated using the formula: ΔTP = TP_T3_ – TP_T0_. ΔTP values were evaluated by comparing them to the perceptibility threshold (PT) of 1.3 and the AT of 4.4 (Paravina, Pérez, and Ghinea 2019).

To perform intraoperative calibration, the operator used a spectrophotometer to measure five specimens at baseline. Thereafter, the same operator repeated the measurement after a week to ensure that the L*, a*, and b* values were not statistically different by using the kappa statistic at a strong level of agreement (value of Kappa > 0.8).

### 2.3 Coffee Thermocycling

All specimens were thermally aged using a thermocycling machine with coffee solution (TC301; King Mongkut's Institute of Technology Ladkrabang, Thailand) at temperatures ranging from 5°C to 55°C, with 30 s of soaking time and 10 s of transfer time, as per ISO 11405 guidelines (ISO‐ISO/TR 11405,405 1994).

The coffee solution (Nescafe red cup; Chachoengsao, Thailand) was made using a filter coffee machine. According to the manufacturer's instructions, a ratio of 1 tablespoon of coffee to 180 mL of water was used. The coffee solution in both containers was replaced every 24 h. Aging processes were carried out for 10,000 thermocycles (T1), which is equivalent to 1 year of clinical use, for 20,000 thermocycles (T2), which is equivalent to 2 years of clinical use, and for 30,000 thermocycles (T3), which is equivalent to 3 years of clinical use (Gale and Darvell 1999).

### 2.4 Surface Roughness

Concerning surface topography of all test materials, surface roughness measurement was made on the test side of the specimens (glazed surface) before (baseline) and after 30,000 cycles of coffee thermocycling using contact‐type surface roughness instruments (Talyscan 150 with stylus for contact 3D scanning; Taylor Hobson Limited, Leicester, UK). The measurement values were taken for the arithmetical roughness mean (Ra).

To ensure standardization, each specimen was evaluated for surface roughness before testing, ensuring a mean surface roughness of less than 0.1 μm.

### 2.5 Scanning Electron Microscope (SEM)

To identify the surface characteristics of specimens, the samples were examined using a high‐energy electron beam while being scanned using a scanning electron microscope (JSM‐5410LV; JEOL, USA). Specimens were coated in a sputtering device with an ultra‐thin layer of electrically conductive gold material and were scanned before and after 30,000 cycles of coffee thermocycling.

### 2.6 Statistical Analysis

Statistical analyses were performed using a statistical software program (IBM SPSS Statistics, v.21 for Windows; IBM Corp). Results of the Kolmogorov–Smirnov test determined that the data were normally distributed (*p* < 0.05). Independent samples *t*‐test was used to compare the means of Δ*E* and ΔTP between two types of materials. One‐way repeated‐measures analysis of variance (ANOVA) followed by Tukey honestly significant difference (HSD) test were performed at a pre‐set alpha of 0.05, meaning that a significant difference exists with 95% confidence to evaluate the association of TP and surface roughness (Ra) of the specimens (*p* < 0.05).

## 3 Results

### 3.1 Color Stability

**Figure 2:**
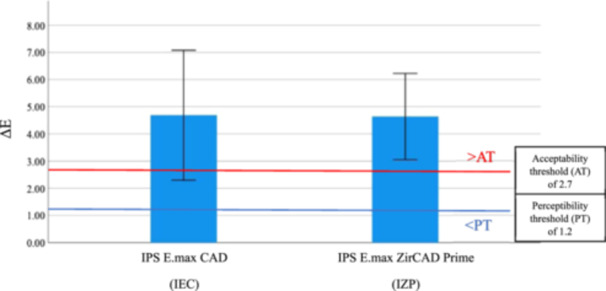
Comparison of color difference values (Δ*E*) between IEC and IZP from baseline to 30,000 cycles of coffee thermocycling. Blue and red horizontal lines represent the perceptibility threshold, PT, and acceptability threshold, AT, respectively.

The results revealed that the mean of color difference (Δ*E*) values of IEC and IZP were 4.69 and 4.64, respectively. The independent samples *t*‐test showed no significant difference in Δ*E* (*p* = 0.202) between IEC and IZP (Table 2). However, both materials showed that the mean of Δ*E* values were higher than the acceptable range (1.2 < Δ*E* ≤ 2.7) (Paravina, Pérez, and Ghinea 2019) after 30,000 cycles of coffee thermocycling (Figure 2).

**Table 2:** Result of mean color change (Δ*E*) and translucency parameter change (ΔTP) of lithium disilicate glass ceramic and high translucent zirconia after 30,000 cycles of coffee thermocycling.

	Δ*E*		ΔTP	
Materials	Mean	SD	Mean	SD
IPS E.max CAD (IEC)	4.69	±2.86	3.25	±1.57
IPS E.max ZirCAD Prime (IZP)	4.64	±1.90	0.63	±0.93
*p* value	0.202		0.089	

### 3.2 TP and Translucency Difference (ΔTP)

The one‐way ANOVA results indicated that there was a significant difference in the TP values across each time interval. Tukey post hoc test showed that IEC had a significant increase in TP (*p* < 0.001) from baseline (T0) to 10,000 cycles (T1), from baseline (T0) to 20,000 cycles (T2), and from baseline (T0) to 30,000 cycles (T3), while IZP showed no significant change at each time interval (*p* = 1.000) (Table 3).

**Table 3:** One‐way ANOVA result of translucency parameter (TP) of lithium disilicate glass ceramic and high translucent zirconia after 30,000 cycles of coffee thermocycling.

	Baseline		10,000 cycles		20,000 cycles		30,000 cycles		
	T0		T1		T2		T3		
Source	Mean	SD	Mean	SD	Mean	SD	Mean	SD	*p* value
IPS E.max CAD (IEC)	16.113^ABC^	±1.73	18.950^A^	±0.73	19.863^B^	±1.01	19.363^C^	±1.00	<0.001*
IPS E.max ZirCAD Prime (IZP)	11.675	±0.55	11.712	±0.34	11.575	±1.06	11.675	±0.89	1.000

*Note:* The same capital letters indicate significant difference within the same row of all materials. A: significant difference between T0 and T1, B: significant difference between T0 and T2, C: significant difference between T0 and T3.

Abbreviation: ANOVA, analysis of variance.

* Tukey post hoc test at a significant level of *p* < 0.05: statistically significant.

**Figure 3:**
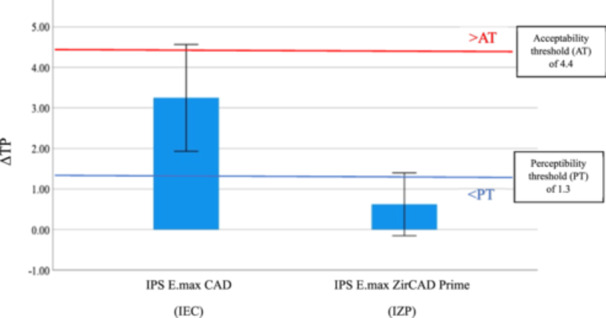
Comparison of the difference in translucency parameter (ΔTP) between IEC and IZP from baseline to 30,000 cycles of coffee thermocycling. Blue and red horizontal lines represent the perceptibility threshold, PT, and acceptability threshold, AT, respectively.

The mean difference in translucency of IEC between TP after 30,000 cycles (TP_T3_) and TP at baseline (TP_T0_) was 3.25 (Table 2), which was within the acceptable range (1.3 < ΔTP ≤ 4.4), indicating an acceptable match in translucency (Paravina, Pérez, and Ghinea 2019). The mean difference in translucency between TP_T3_ and TP_T0_ of IZP was 0.63 (Table 2), which was below the perceptibility threshold, indicating an excellent match in translucency (Paravina, Pérez, and Ghinea 2019) (Figure 3).

### 3.3 Surface Roughness

The outcome of the one‐way ANOVA showed a significant difference in Ra values between baseline and after subjecting the test specimens to 30,000 cycles of coffee thermocycling. The Tukey post hoc test demonstrated a significant increase in surface roughness for IZP (*p* = 0.007) after 30,000 cycles of coffee thermocycling, while IEC showed no significant difference (*p* = 0.134) (Table 4).

**Table 4:** One‐way ANOVA result of mean surface roughness (Ra) of lithium disilicate glass ceramic and high translucent zirconia after 30,000 cycles of coffee thermocycling.

	Baseline	30,000 cycles	
Source	Mean	Mean	*p* value
IPS E.max CAD (IEC)	0.038 µm	0.039 µm	0.134
IPS E.max ZirCAD Prime (IZP)	0.040 µm	0.043 µm	0.007*

Abbreviation: ANOVA, analysis of variance.

* Tukey post hoc test at a significant level of *p* < 0.05: statistically significant.

### 3.4 SEM Photomicrographs

**Figure 4:**
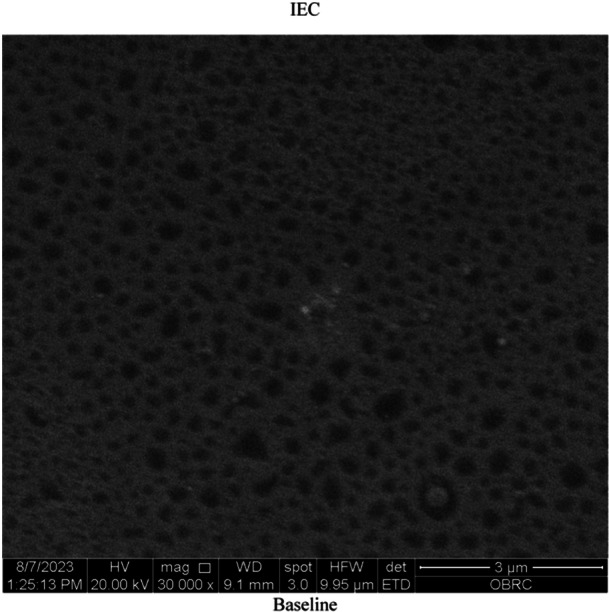
Scanning electron microscope photomicrograph (magnification ×30,000) demonstrates the surface of IEC at the baseline.

**Figure 5:**
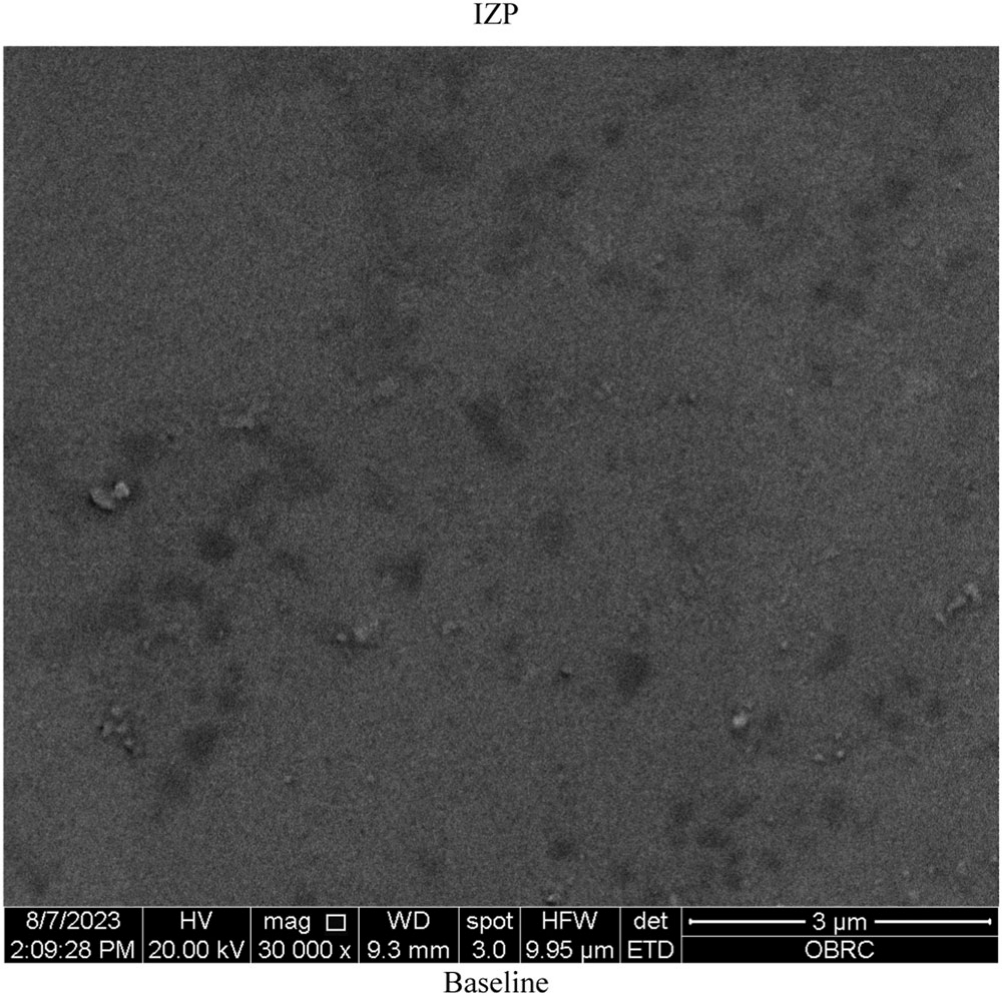
Scanning electron microscope photomicrograph (magnification ×30,000) demonstrates the surface of IZP at the baseline.

**Figure 6:**
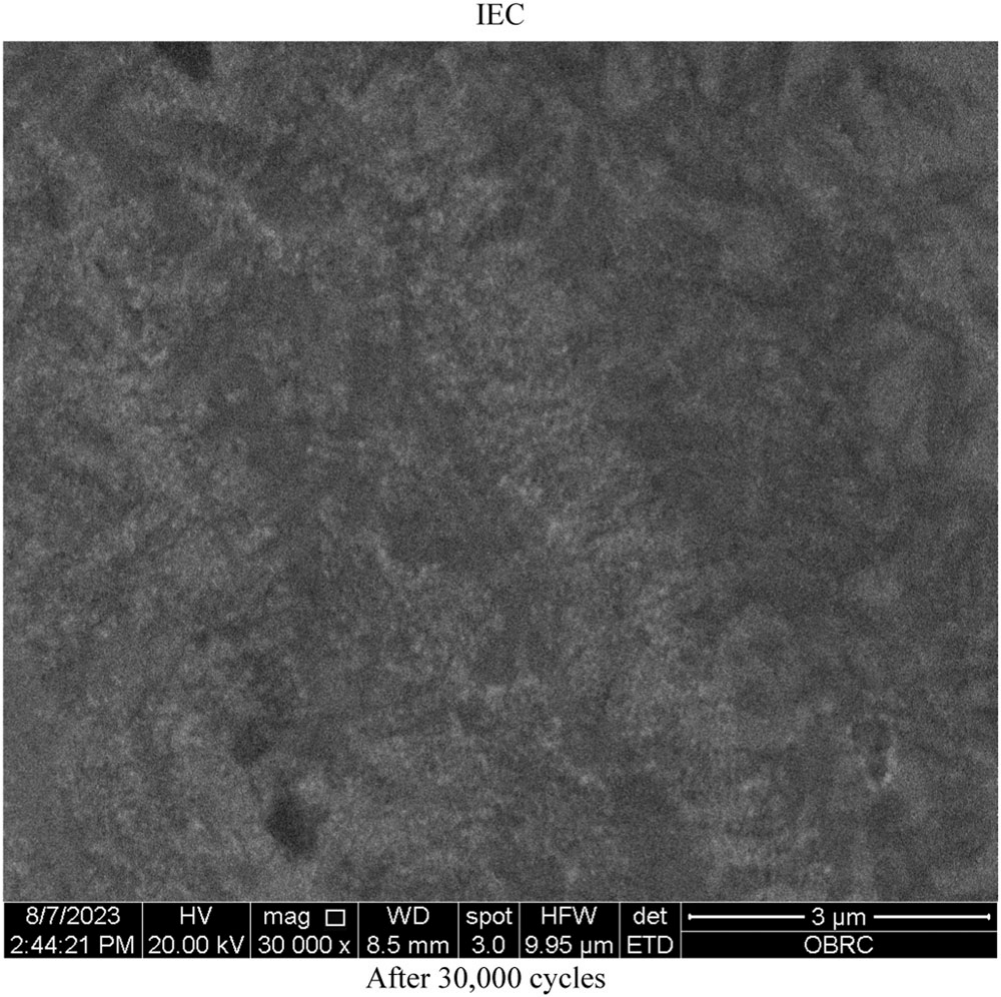
Scanning electron microscope photomicrograph (magnification ×30,000) demonstrates the surface of IEC following 30,000 cycles of coffee thermocycling.

**Figure 7:**
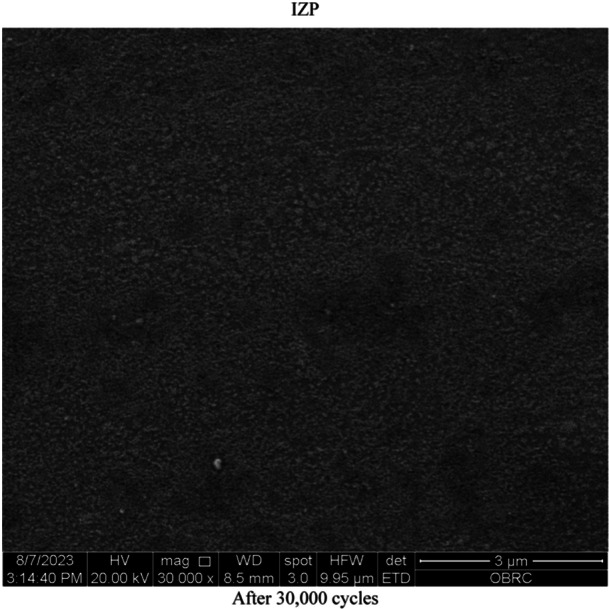
Scanning electron microscope photomicrograph (magnification ×30,000) demonstrates the surface of IZP following 30,000 cycles of coffee thermocycling.

SEM photomicrographs (magnification ×30,000) of IEC specimen at baseline demonstrated regular surface morphology and numerous surface porosities (Figure 4); IZP demonstrated regular surface morphology at baseline (Figure 5). After 30,000 cycles of coffee thermocycling, SEM photomicrographs of IEC specimen showed a thickened layer of particle staining on the test surface and less numerous surface porosities (Figure 6). The test surface of IZP appeared to have a thickened layer of particle staining on the surface (Figure 7) compared to its baseline surface.

## 4 Discussion

For color stability, the results of coffee thermocycling after 30,000 cycles displayed that all materials had mean values of Δ*E* beyond the AT for color differences (Figure 2). Therefore, the first null hypothesis was rejected, and this study found that there was no significant difference in color stability between IEC and IZP (*p* = 0.202). Within this study design, it could imply that the surfaces of all ceramic materials can become stained in a similar manner.

Surface roughness is an important factor that leads to external staining of surface materials (Vasiliu et al. 2020). Glazing can provide a glossy surface and a smooth ceramic surface by sealing any surface holes found within ceramics (McLean 1991). Therefore, a properly glazed ceramic surface provides stain‐resistant properties (Chotipanvidhayakul et al. 2021). Another factor that can influence on stain adherence is the glaze material itself. Difference in chemical component of ceramic glazing material yields different surface free energy of ceramic surface. Water tends to disperse effectively across ceramic surfaces with high surface free energy, displacing oil substances to separate from ceramic surface when in contact with water (Liang et al. 2006). A study investigated the cleanability of ceramic glazes and reported that the greater the smoothness and surface free energy, the more effective the cleanability of the ceramic material (Wang et al. 2014). This could explain the color difference observed in both glazed ceramics in our study after 30,000 thermocycles in coffee. The mean surface roughness values of both ceramic types after undergoing thermocycling were less than 0.1 μm, indicating that the surfaces remained very smooth. Despite a significant increase in surface roughness being specific to the IZP group, the IEC group also demonstrated comparable susceptibility to staining.

A previous study reported that thermocycling leads to an increase in the surface roughness of both lithium disilicate and zirconia, and no correlation was found between color change and surface roughness (Yuan et al. 2018). Additionally, a recent study revealed that thermocycling could potentially affect glazed surfaces, particularly for the zirconia‐reinforced lithium silicate ceramic, causing an elevation in surface roughness of the materials (Vasiliu et al. 2020). The surface roughness results in this study suggest that there may be changes in the glazed surface of the IZP group during thermocycling. These changes lead to an increase in surface roughness which is consistent with findings in abovementioned studies.

**Figure 8:**
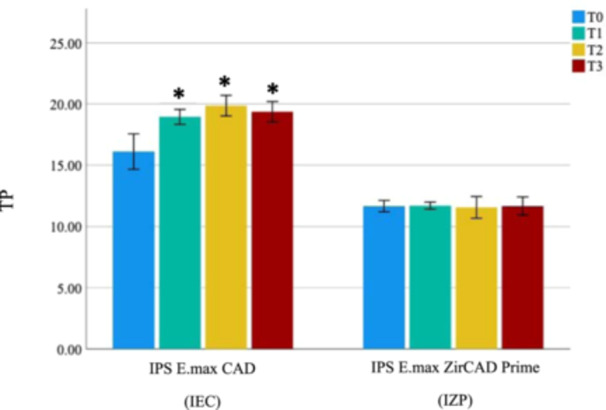
Comparison of the mean translucency parameter (TP) between IEC and IZP from baseline to 30,000 cycles of coffee thermocycling. T0, at baseline; T1, after 10,000 thermocycles; T2, after 20,000 thermocycles; T3, after 30,000 thermocycles (T3); *Tukey post hoc test at a significant level of *p* < 0.05: statistically significant.

For TP, the second null hypothesis was partially rejected because the results showed a statistically significant difference between TP of the IEC (*p* < 0.001) at T0 to T1, T0 to T2, and T0 to T3. Whereas there was no statistically significant difference between TP of the IZP at T0 to T1, T0 to T2, and T0 to T3 (Figure 8). This result resembled the previous study, which reported that lithium disilicate glass ceramic exhibited a significant increase in TP values after thermocycling (Aljanobi and Al‐Sowygh 2020).

In a prior study, it was noted that the primary factor influencing the translucency of ceramics is surface porosity; as surface porosities increase, ceramics become opaquer. Every time it occurs, surface porosity reduces TP values and has a negative effect on light scattering (Wang, Zhang, and Luo 2013). The results of this current research were finding that after 30,000 cycles of coffee thermocycling, the surface of an IEC (Figure 6) demonstrated considerably lesser surface pores than at baseline (Figure 4). As a result, the TP value in the IEC was significantly increased after coffee thermocycling.

Low‐temperature degradation (LTD) refers to the spontaneous tetragonal to monoclinic (t‐m) phase transformation that occurs over time at low temperatures in aged zirconia. LTD results in increased wear rates and surface roughness due to the loss of small zirconia grains from the material's surface (Lughi and Sergo 2010). A study assessed the translucency of different thicknesses of translucent zirconia, revealing that aging had a significant effect on the TP of zirconia specimens with a thickness of 0.5 mm compared to before aging, while the thicknesses of 0.8, 1.0, and 1.2 mm were not significantly affected. This can be elucidated by the notion that thicker specimens possess a greater number of zirconia grains per unit volume and a higher zirconia percentage compared to thinner specimens. Hence, thin portions are more significantly affected by LTD‐induced transformations in the microstructure (Abdelbary et al. 2016). This may be the most accepted explanation for the increased scattering centers and decreased transmittance of aged zirconia. Our study utilized 1.0 mm thickness of ceramic specimen and found no statistically significant difference between TP of the IZP after 30,000 cycles of coffee thermocycling. This finding aligns with the earlier mentioned study, which indicates a correlation between the thickness of the zirconia specimen and the observed results, and aligns with the possibility that the aging process may primarily impact only the superficial layer of the zirconia (Wang, Zhang, and Luo 2013).

In this study, a coffee solution was used instead of a water solution during thermocycling, resulting in the materials' surfaces being exposed to coffee for 24 h a day. However, people typically consume coffee for an average of 1 h per day and are commonly exposed to their routine brushing for oral prophylaxis. Therefore, Δ*E* values in this experiment might be overestimated for a 3‐year consecutive time interval of daily coffee drinking.

Recently, a study found that the glazed ceramic surface exhibited higher surface roughness compared to the polished ceramic surface both before and following thermocycling (Diana Vasiliu, Dragoș Uţu, and Porojan 2021). This finding suggests the need for further research to explore additional surface treatments as the study intervention.

The present study shows that the new high translucent zirconia materials exhibit optical properties that outperform lithium disilicate glass ceramic in terms of translucency stability while demonstrating comparable performance in color stability. In clinical practice, using both types of ceramic materials together for esthetic purposes is acceptable. However, it is essential to consider that the aging process may influence the translucency discrepancy between these two materials.

## 5 Conclusions

Within the limitations of this current study, it was concluded that coffee thermocycling affected the color stability of all materials. However, for TP, only lithium disilicate glass ceramic was influenced by coffee thermocycling. High translucent zirconia had superior translucency stability compared to lithium disilicate glass ceramic.

## Author Contributions

**Ratchaphat Khomprang:** conceptualization, methodology, investigation, results, writing the original draft, writing–review and editing, project administration. **Jeerapa Sripetchdanond:** conceptualization, methodology, results, writing the original draft, writing–review and editing, project administration, supervision. **Wareeratn Chengprapakorn:** conceptualization, methodology, results, writing the original draft, project administration, supervision.

## Ethics statement

The authors have nothing to report.

## Conflicts of Interest

The authors declare no conflicts of interest.

## Data Availability

The data sets utilized in this investigation can be obtained from the corresponding author upon reasonable request.
